# Evidence that ERF transcriptional regulators serve as possible key molecules for natural variation in defense against herbivores in tall goldenrod

**DOI:** 10.1038/s41598-020-62142-4

**Published:** 2020-03-24

**Authors:** Kento Takafuji, Hojun Rim, Kentaro Kawauchi, Kadis Mujiono, Saki Shimokawa, Yoshino Ando, Kaori Shiojiri, Ivan Galis, Gen-ichiro Arimura

**Affiliations:** 1 Department of Biological Science and Technology, Faculty of Industrial Science and Technology, Tokyo University of Science, Tokyo, 125-8585 Japan; 2 Institute of Plant Science and Resources (IPSR), Okayama University, Kurashiki, 710-0046 Japan; 3 Faculty of Agriculture, Mulawarman University, Samarinda, 75119 Indonesia; 4 Field Science Center for Northern Biosphere, Hokkaido University, Sapporo, 060-0809 Japan; 5 Faculty of Agriculture, Ryukoku University, Otsu, 520-2194 Japan

**Keywords:** Plant ecology, Herbivory

## Abstract

We collected *Solidago altissima* clones to explore their leaf damage resistance, and as a result identified five accessions that exhibited variable defense abilities against the generalist herbivore *Spodoptera litura*. In order to characterize molecules involved in such natural variation, we focused on ethylene response factors (ERFs) that exhibited distinct transcription patterns in the leaves of the five accessions (e.g., S1 and S2) after wounding: the transcript of *SaERF1* and *SaERF2* was induced in wounded S1 and S2 leaves, respectively. Although transcription levels of *SaERFs* in leaves of the five accessions did not correlate with the accessions’ phytohormone levels, these transcription levels accorded with the possibility that ethylene and jasmonate signaling play crucial roles in wound-induced transcription of *SaERF1* in S1 leaves, and *SaERF2* in S2 leaves, respectively. SaERF1 was found to be a positive regulator of the GCC box and DRE element in the upstream regions of promoters of defense genes, whereas SaERF2 served as a negative regulator of genes controlled through the GCC box. Transgenic Arabidopsis plants expressing *SaERF1* or *SaERF2* showed enhanced and suppressed transcript levels, respectively, of a defensin gene, indicating that ERFs may be partly responsible for herbivore resistance properties of *S. altissima* accessions.

## Introduction

Intraspecific variations of defense traits in cultivated and wild plants play important roles in ecosystems. For example, the variation of direct defense chemicals (glucosinolates and 3-butenyl glucosinolates) in several cultivated and wild cabbage populations contributes to the differences of the performance of a herbivorous arthropod on them^1^. Moreover, variations in the emission of volatile compounds from plants according to their genotypes affect volatile-mediated communications with neighboring sagebrush plants^2^ and the attraction of predatory mites towards *Tetranychus urticae*-infested common bean plants^3^.

The tall goldenrod (*Solidago altissima* L. [Asteraceae]) was introduced into Japan from North America as an ornamental plant more than a century ago, and nowadays dominates many wild ecosystems throughout Japan. *S. altissima* emerges from overwintering rhizomes as the ground warms in April, and its shoots grow continuously until September. Flowering occurs from late October to November. Although aboveground shoots disappear in winter, rhizome connections persist for up to 5–6 years^4^. This species has been intensively used to study the impact of genotypic variation on ecosystem dynamics. For instance, it has been demonstrated that genetic variation of *S. altissima* affects the population dynamics of the aphid *Uroleucon nigrotuberculatum* Olive, a specialist herbivore^5–8^, and various other herbivore species^9^. Notably, the degree of genetic similarity among *S. altissima* genotypes is highly relevant to the similarity of the characteristics of the herbivore community: for instance, the defensive properties of *S. altissima* against aphids are linked with their genetic background^10^. Although numerous studies have been conducted concerning the ecology, biodiversity, and evolution of the genetic variation of *S. altissima* in connection to its plant defense traits, little is known about the molecular bases of these defense traits.

In the current study, in order to identify genes that are potentially responsible for the different defense abilities of different *S. altissima* genotypes (accessions), we first collected *S. altissima* clones from various sites that had similar environmental conditions in the temperate zone, and we used two representative clones for preliminary RNA-sequencing (RNA-Seq) analysis of their gene expression profiles when they were subjected to mechanical leaf damage. The results revealed that a large array of transcription factor (TF) genes were differentially regulated between damaged leaves of these two clones, with ethylene response factors (ERFs) playing dominant roles in this differential regulation. ERFs are large plant-specific APETALA2/ETHYLENE RESPONSE FACTORs (AP2/ERFs) involved in plant stress responses in an array of plant taxa^11^. We focused on ERFs because they are involved in defense responses to biotic stresses^12–14^. For instance, a mutant plant of JRE4 (an ERF) exhibits increased susceptibility to the generalist herbivore *Spodoptera litura*^13^. However, little has been reported about the significance of various ERFs in herbivore resistance, and even less has been reported on the genetic variation of plants’ resistance to herbivores. Here, we present the molecular functions, transcriptional regulation, and potential roles of two ERFs (SaERF1 and SaERF2) in plant defense responses in *S. altissima* foliage, and we discuss the contribution of SaERFs to the differing defense abilities of different *S. altissima* genotypes.

## Results

### Isolation and phenotypic characterization of *S. altissima* accessions

**Figure 1:**
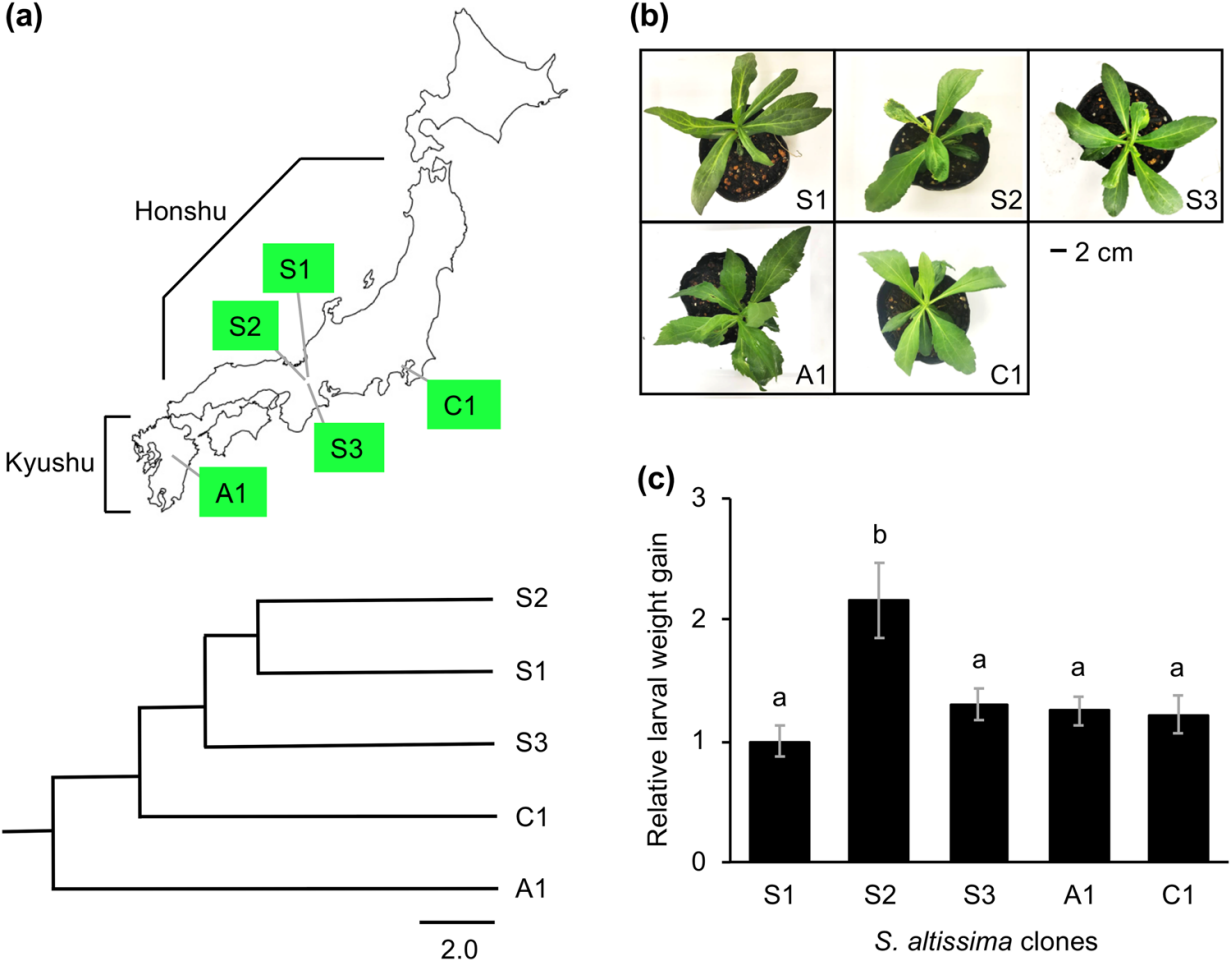
Genetic diversity of *Solidago altissima* clones in relation to defense property. (**a**) A map for collection sites of *S.altissima* clones (S1, S2, S3, A1 and C1), and the clones’ phylogenic tree. The map shows the main islands of the Japanese archipelago, including Honshu and Kyushu islands. The clones’ phylogenic tree was determined based on amplified fragment length polymorphisms (AFLPs). (**b**) The phenotype of seedling plants grown from rhizomes in soil for about 4 weeks. (**c**) Defense property of *Solidago altissima* clones. The net body weight that *Spodoptera litura* larvae gained during 4 days after they had been placed on potted plants of *Solidago altissima* clones. Data represent the mean and standard error (*n* = 16–18). The means indicated by different small letters are significantly different based on an ANOVA with post hoc Tukey’s HSD (*P* < 0.05).

We isolated *S. altissima* clones at distinct locations in the temperate zone. Clones S1, S2 and S3 were isolated proximately to each others’ habitat. Clones A1 and C1 were isolated further away, at a site 600 km southwest, and at a site 400 km east of the S1-3 site, respectively, in Honshu and Kyushu Islands of Japan. Their relative genetic distances, determined by amplified fragment length polymorphism (AFLP) analysis, appeared to be linked to their original geographic distances (Fig. 1a).

Growth and development of seedlings after propagation from rhizomes and the photosynthetic efficiency of the five clones were not significantly different (Fig. 1b and Supplemental Fig. 1). However, these clones exhibited variable defense properties against larval development of the generalist herbivore *Spodoptera litura* on potted plants. *S. litura* larvae applied onto potted S2 plants showed the greatest weight gain during 4 days, compared to that on the plants with the other four accessions assessed (Fig. 1c). S1 gained marginally less weight during 4 days, compared to the weight gain on the plants with the other four accessions assessed, but note that the difference was not significant (*P* > 0.05, one-way ANOVA with Tukey’s HSD test) (Fig. 1c).

### Mining and isolation of *S. altissima ERF* clones

To mine and characterize the possible molecules that might contribute to natural variation of defense ability, we performed preliminary RNA-Seq analysis of mRNA in mechanically damaged leaves of S1 and S2 *S. altissima* clones. The transcriptome revealed that a large array of TF genes, including 3 *WRKY*, a *MYB*, a *bHLH91*, and 42 *SaERF* genes, among a total of 19679 Unigenes/Contigs, were expressed differently between these clones in the damaged leaves (Supplemental Table 1). Of them, we especially focused on 6 ERFs with particularly different expression between clones (Unigene32270 and Unigene34046 [expressed in damaged S1 leaves alone] and Unigene24680, Unigene24674, Unigene23379 and CL2733.Contig1 [expressed in damaged S2 leaves alone]) (Supplemental Table 2). We therefore explored the full-length open reading frame (ORF) sequences of the predicted ERF genes, except for Unigene34046, which was found as a chimeric gene, and CL2733.Contig1, which had already been annotated as covering the full-length ORF. Finally, we recovered a full-length cDNA of Unigene32270 by genome inverse PCR and rapid amplification of cDNA ends (RACE). Subsequently, Unigene32270 and CL2733 were annotated as *SaERF1* and *SaERF2*, and are so referred to hereafter.

### Possible factors involved in *SaERF1* and *SaERF2* transcriptional variations

**Figure 2:**
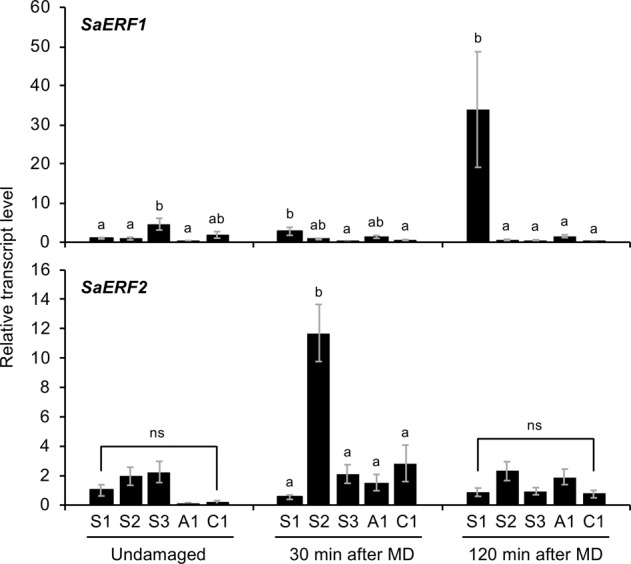
Transcriptional patterns of *SaERF1* and *SaERF2* in undamaged leaves and leaves at 30 min or 120 min after mechanical damage (MD) in various *S. altissima* clones. Relative transcript abundances were determined after normalization of raw signals with the abundance of the housekeeping transcript of a histone gene (CL2599.Contig7). Data represent the means and standard errors (*n* = 6–10). The means indicated by different small letters are significantly different based on an ANOVA with post hoc Tukey’s HSD (*P* < 0.05). ns, not significant.

**Figure 3:**
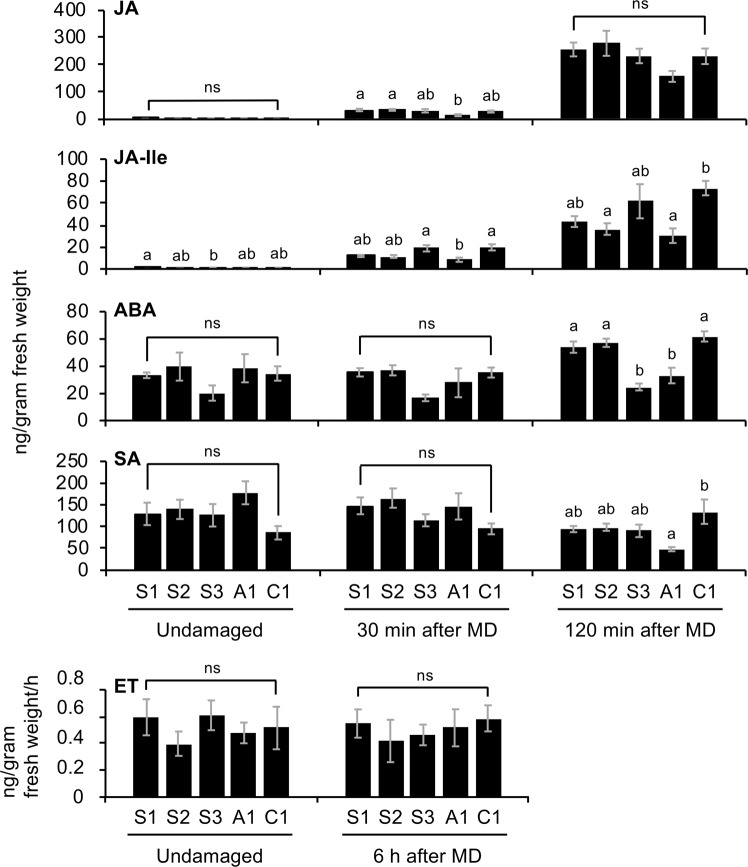
Foliage phytohormone levels. Endogenous levels of jasmonic acid (JA), jasmonoyl-L-isoleucine (JA-Ile), abscisic acid (ABA), and salicylic acid (SA) in undamaged leaves and leaves 30 min and 120 min after MD treatment. Ethylene (ET) levels in the headspace of the cut leaflets incubated with or without subsequent MD treatment for 6 h were determined. Data represent the means and standard errors (*n* = 4–6). The means indicated by different small letters are significantly different based on an ANOVA with post hoc Tukey’s HSD (*P* < 0.05). ns, not significant.

**Figure 4:**
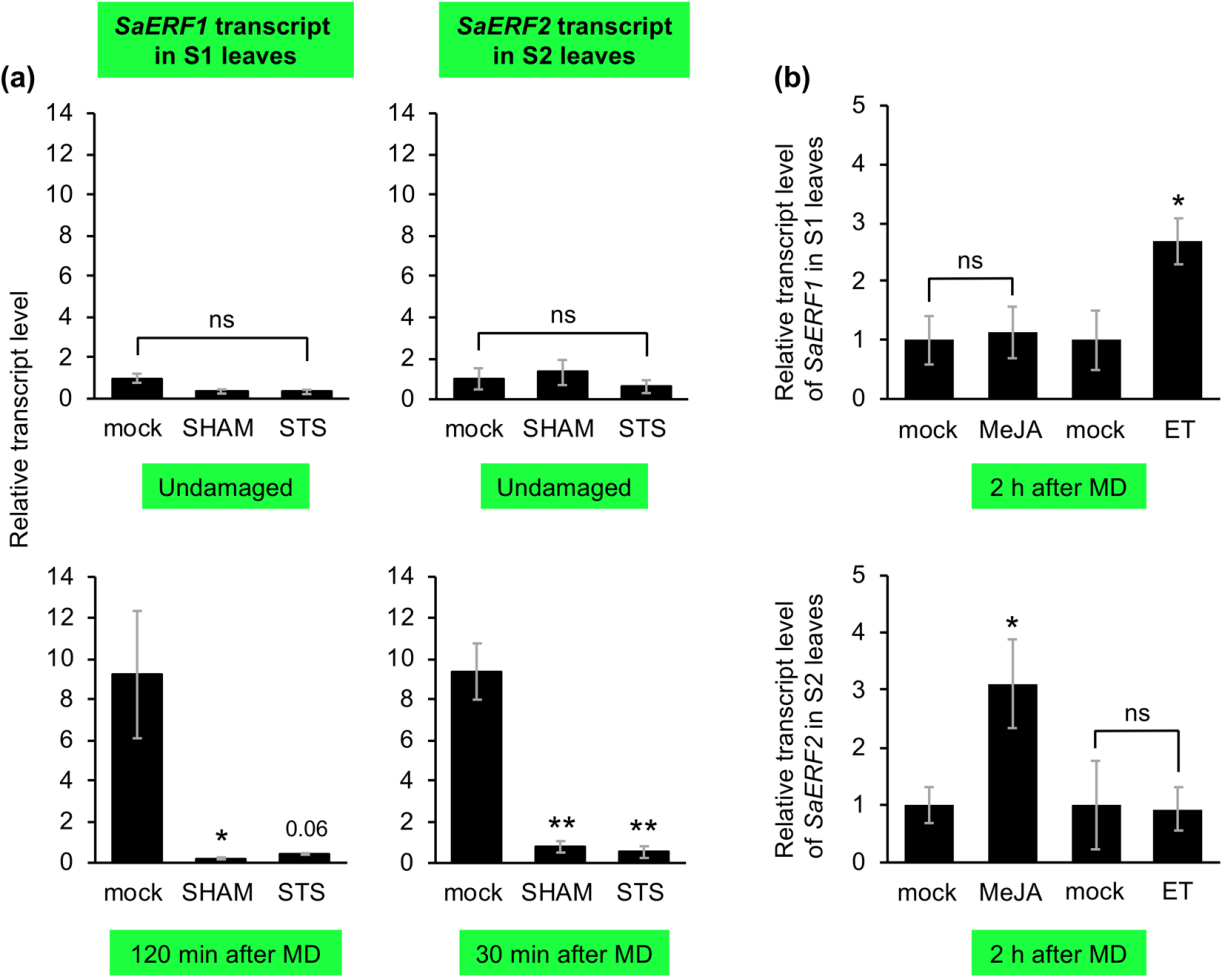
Involvement of jasmonate and ethylene signaling in transcriptional regulation of *SaERF*. (**a**) Leaves were pretreated with aqueous solution (mock), salicylhydroxamic acid (SHAM) or silver thiosulfate (STS). Transcript accumulation levels of *SaERF1* and *SaERF2* in undamaged S1 and S2 leaves and leaves 120 min and 30 min after mechanical damage (MD), respectively, were determined. Data represent the mean ± standard error (n = 4–5). Data marked with an asterisk are significantly different from those of mock treatment, based on an ANOVA with Holm’s sequential Bonferroni post-hoc test (**, 0.001 ≤ *P* < 0.01; *, 0.01 ≤ *P* < 0.05). Otherwise, the mean followed by a *P*-value is marginally different. (**b**) Transcript accumulation levels of *SaERF1* and *SaERF2* in response to exogenous application of methyl jasmonate (MeJA) solution (0.5 mM) or ethylene (ET) gas (1 ppm) for 120 min. Relative transcript abundances were determined after normalization of raw signals with the abundance of the housekeeping transcript of a histone gene (CL2599.Contig7). Data represent the mean ± standard error (*n* = 4–5). Data marked with an asterisk are significantly different based on an ANOVA from the respective of mock treatment (*, 0.01 ≤ *P* < 0.05). ns, not significant.

It was found that *SaERF1* was highly expressed only in S1 leaves at 120 min after MD, while *SaERF2* was highly expressed only in S2 leaves after 30 min (Fig. 2). Since the expression of *ERF*s is generally regulated through a suite of phytohormone signalings^15,16^, the levels of accumulation of endogenous jasmonates (jasmonic acid [JA] and JA’s active form [JA-Ile]), abscisic acid (ABA), salicylic acid (SA) and ethylene in leaves of the five *S. altissima* accessions were determined. In summary, neither the endogenous level of jasmonates, ABA or SA, nor ethylene emission of undamaged or damaged leaves, differed among these five accessions (Fig. 3). We therefore explored whether jasmonate and ethylene signaling might be specifically involved in the transcriptional activation of *SaERF1* and *SaERF2* in S1 and S2, respectively, as these hormones are the most prominent regulators of *ERF* expression during plant stress responses^14^. We assessed their involvement by using SHAM, a jasmonate synthesis inhibitor^17,18^ and STS, an ethylene response inhibitor^19^. We found that pretreatment of S1 or S2 leaves with SHAM or STS decreased *SaERF1* and *SaERF2* expression levels after MD, respectively (Fig. 4a). Moreover, treatment of leaves with methyl jasmonate (MeJA) solution or ethylene gas led to higher transcriptional levels of *SaERF1* in S1 leaves and *SaERF2* in S2 leaves, respectively (Fig. 4b), indicating that S1 and S2 responded differently to these essential defense regulators.

### Molecular function of SaERFs

Based on the deduced amino acid sequences of these SaERFs, we predicted that these SaERFs belong to distinct groups of ERFs (SaERF1, group IX; SaERF2, group VIII) (Supplemental Fig. 2)^11^. SaERF2 has a CMII-2 repressor motif (DLNxxP), which is frequently present in the C-terminal region of ERF group IIa^11^, and is also present in the N-terminal region of a novel B3 domain repressor protein (Supplemental Fig. 3)^20^.

**Figure 5:**
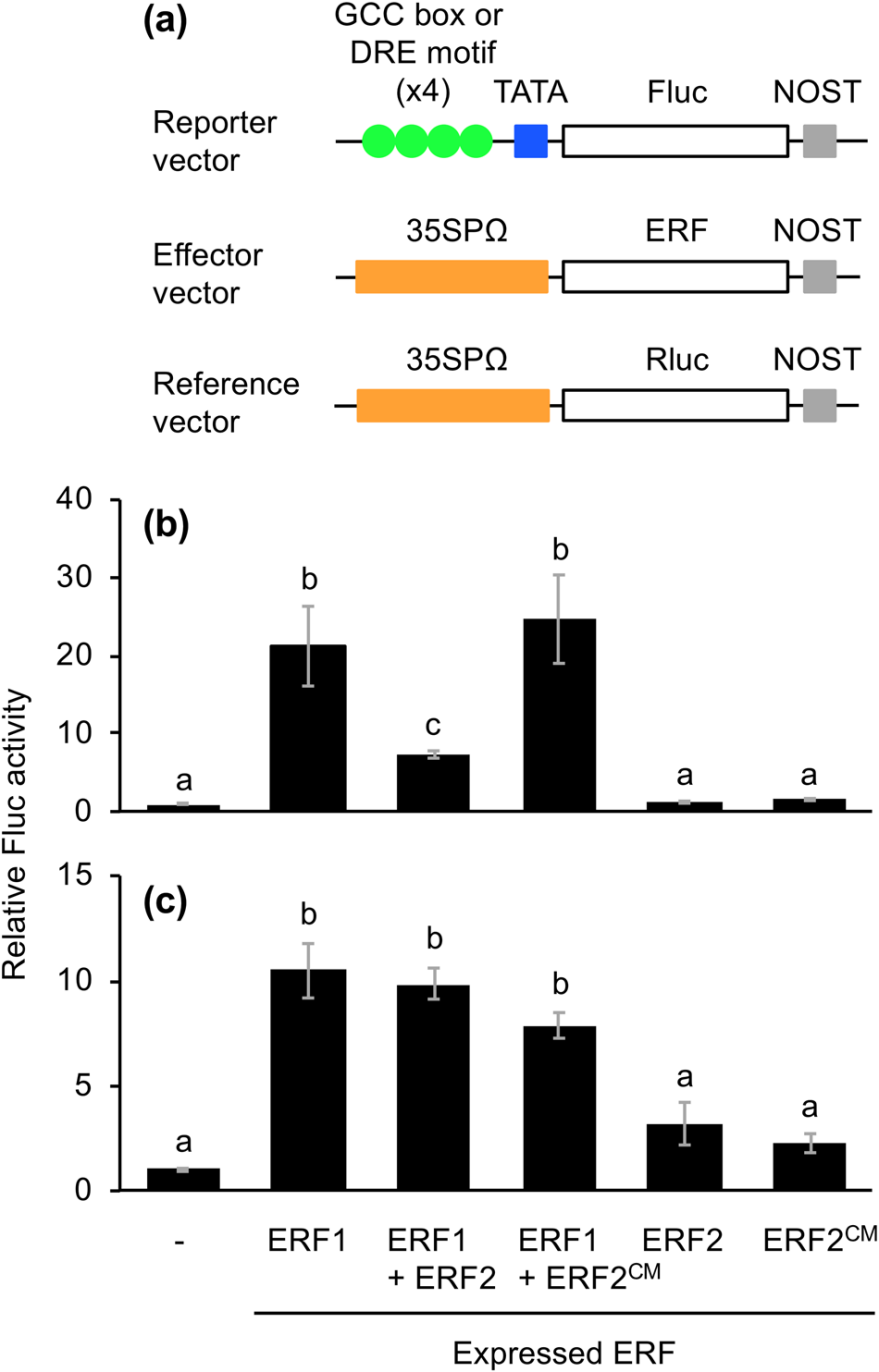
Dual luciferase activity mediated through SaERF associated with GCC box and DRE element in the promoter. (**a**) Schematic diagram of the reporter and effector vectors used in dual luciferase assays. The firefly luciferase (Fluc) gene under control of a 4x GCC box (**b**) or 4x DRE element (**c**) sequence was fused to a minimal TATA-box and coexpressed with or without (−) a gene for SaERF1 (ERF1), SaERF2 (ERF2) or ERF2 mutant deficient in CMII-2 repressor motif (ERF2^CM^), or a mixture of ERF1 and ERF2 or ERF1 and ERF2^CM^ in Arabidopsis protoplast cells. To account for the efficiency of transformation, Fluc activity produced due to the transfected reporter construct was expressed as the value normalized by the Renilla luciferase (Rluc) activity produced due to the co-transfected reference vector. Data represent the means and standard errors (*n* = 3). The means indicated by different small letters are significantly different based on an ANOVA with post hoc Tukey’s HSD (*P* < 0.05). 35 SPΩ, cauliflower mosaic virus 35S promoter with Ω translation enhancer; NOST, *nopaline synthase* terminator; TATA, TATA-box.

To investigate the molecular function of these SaERFs, each (or both) of the SaERFs was expressed using a vector for expression of a firefly luciferase (Fluc) reporter gene under the control of a chimeric promoter that consisted of four inverted repeats of the GCC box (ERF-binding *cis*-element^21^) fused to a minimal TATA-box, in Arabidopsis mesophyll protoplasts (Fig. 5a). Expression of SaERF1 caused a 21-fold increase of Fluc activity in comparison to the activity in the absence of SaERF1 (Fig. 5b). Co-expression of SaERF1 and SaERF2 caused decreased transactivation of Fluc activity in comparison to the transactivation caused by the expression of SaERF1 alone, suggesting that SaERF2 served as a suppressor of SaERF1. Moreover, when an SaERF2 mutant deficient in the CMII-2 repressor motif (SaERF2^CM^) was expressed concomitantly with SaERF1, the Fluc activity was increased to the level achieved by expression of SaERF1 alone. Expression of either SaERF2 or SaERF2^CM^ alone did not cause transactivation of the reporter gene.

The DRE element is another ERF-binding *cis*-regulatory element present in the promoter region of abiotic stress-responsive genes in several plant taxa^22,23^. We therefore explored the effects of SaERF1 and SaERF2 utilizing the transient Fluc expression system in protoplast cells, using a DRE element. We found that the Fluc activity was transactivated by expression of SaERF1 but not SaERF2 (Fig. 5c). The Fluc activity was not decreased by concomitant expression of either SaERF2 or SaERF2^CM^ with SaERF1 in comparison to the activity achieved by expression of SaERF1 alone, indicating that SaERF2 did not act as a suppressor of SaERF1 during DRE-promoted transactivation.

### Defense ability of transgenic Arabidopsis plants expressing *SaERF*

To assess the *in planta* function of SaERFs, we obtained three lines of transgenic Arabidopsis plants expressing *SaERF1* or *SaERF2* under the control of the constitutive cauliflower mosaic virus 35 S promoter (35SP). Two representative lines (ERF1-OX1 and ERF1-OX2 for *SaERF1*) and (ERF2-OX2 and ERF2-OX3 for *SaERF2*) produced significant levels of *SaERF1* and *SaERF2* transcripts, respectively, (Fig. S2) and thus were used for further analyses.

**Figure 6:**
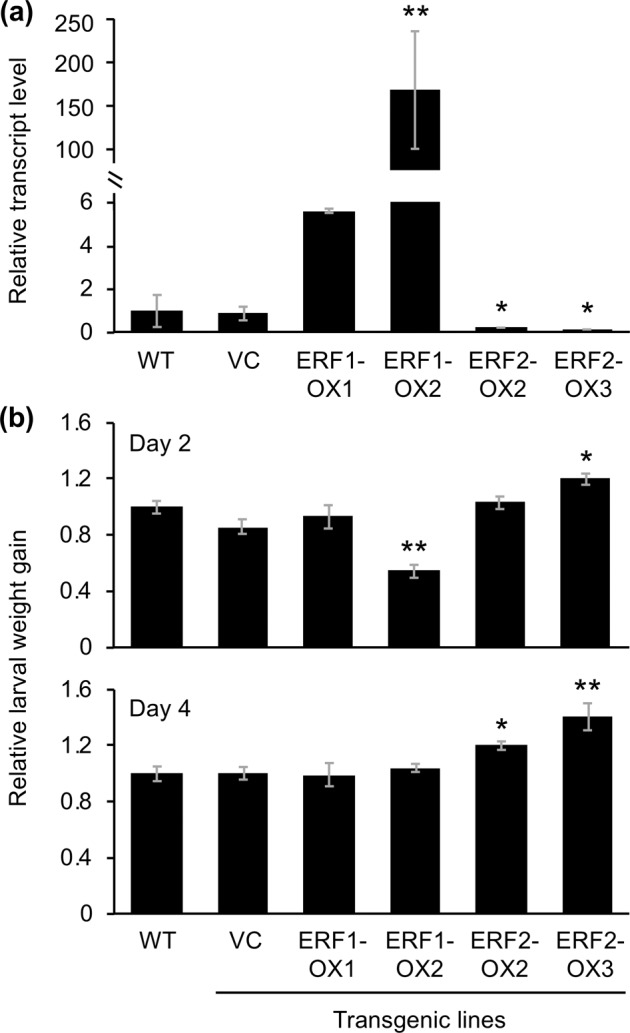
Defense property of *SaERF*-expressing Arabidopsis lines. (**a**) Transcript levels of defensin gene *PDF1.2* in the leaves of wild-type (WT) plants, *SaERF1*-expressing plants (ERF1-OX1 and ERF1-OX2), *SaERF2*-expressing plants (ERF2-OX2 and ERF2-OX3) and vector control (VC) plants. Relative transcript abundances were determined after normalization of raw signals with the abundance of the housekeeping transcript of the Arabidopsis *ACT8* gene (At1g49240). Data represent the mean and standard error (*n* = 5). (**b**) The net body weight that *Spodoptera litura* larvae gained during 2 days or 4 days after they had been placed on potted plants of WT or transgenic lines (VC or SaERF-expressing plants [ERF1-OX1, ERF2-OX2, ERF-OX2, and ERF-OX3]). Data represent the mean and standard error (*n* = 12–15). Data marked with an asterisk are significantly different from those of WT, based on an ANOVA with Holm’s sequential Bonferroni post-hoc test (**, 0.001 ≤ *P* < 0.01; *, 0.01 ≤ *P* < 0.05).

Based on the facts that transcriptional regulation of *PDF1.2*, the Arabidopsis defensin gene, is promoted through the GCC box (located at −255 to −261) and DRE element (located at −612 to −617) in the promoter region^24^, we then analyzed *PDF1.2* expression in the transgenic leaves. ERF1-OX2, which exhibited the highest expression of *SaERF1*, showed a constitutively increased *PDF1.2* expression level in mature leaves (approximately 140-fold the level in wild-type (WT) leaves; Fig. 6a). This accorded with the lower weight gain of larvae of the generalist herbivore *S. litura* grown on the potted plants for 2 days, compared to that on WT plants (Fig. 6b). In contrast, the two lines constitutively expressing *SaERF2* showed lower constitutive levels of *PDF1.2* expression in leaves compared to WT leaves (Fig. 6a). Again, these findings accorded with the higher weight gain of *S. litura* larvae grown on the potted transgenic plants for 2 and/or 4 days, compared to that of larvae grown on WT plants (Fig. 6b).

## Discussion

The nature of intraspecific genetic variations of *S. altissima* that contribute to its environmental adaption has been intensively studied during the last decade^5–8,25^. Although it is clear that herbivory pressure is closely linked to *S. altissima* polymorphism^26,27^, the responsible regulatory factor(s) have not yet been identified. Thus, the present study aimed to examine the possible role(s) of the regulatory factors ERFs in this linkage. ERFs, ones of the largest families of TFs, are partially involved in the genetic, molecular, and metabolic diversity of various plant species and genotypes. For instance, it has been shown that different arrays of ERFs are expressed in two cultivars of Zucchini fruit, cv. Natura (chilling tolerant) and cv. Sinatra (chilling sensitive), in response to chilling injury^28^.

Notably, the expression pattern of the repressor *SaERF2* was related to the susceptibility to herbivores in both S2 plants and transgenic Arabidopsis plants expressing *SaERF2* (Figs. 1c and 6b). In contrast, S1 did not show strong defensive ability against *S. litura* larvae (Fig. 1c), although it abundantly expressed the positive regulator *SaERF1* (Fig. 1d). This might be because SaERF1 is not a key defense regulator, in spite of a possibility suggested by the fact that transgenic Arabidopsis plants expressing *SaERF1* showed enhanced defense against *S. litura* larvae for the initial 2 days but not at 4 days (Fig. 3). Another possibility is that concomitantly activated regulatory factor(s), such as unknown repressors, modulate the defense ability of S1 plants after herbivore attack (Supplemental Tables 1 and 2). Otherwise, SaERF1 may be more responsible for defense against other types of herbivorous pests, such as the aphid *U. nigrotuberculatum* Olive. Taken together, *SaERF1* may function concomitantly with other defense and regulatory genes that are differentially expressed among accessions (Supplemental Table 1), and that partially contribute to the characteristics of defense properties of the respective accessions.

SaERF1 is predicted to belong to ERF group IX (Supplemental Fig. 2), whose members share characteristics of the CMIX motifs (CMIXs 1, 3 and 4), putative acidic regions that might function as transcriptional activation domains^11,21^. In contrast, SaERF2 acts as a competitive repressor against other activators, including SaERF1, regarding GCC box-promoted transactivation (Fig. 2b). However, SaERF2 does not have a typical ERF-associated amphiphilic repression (EAR) motif, the conserved sequence [(L/F)DLN(L/F)xP] present in the C-terminal regions of repressor-type ERF proteins^21,29^. The CMII-2 repressor motif (DLNxxP), which is a novel B3 domain in a repressor protein^20^, and is frequently present in the C-terminal region of ERF group IIa proteins^11^, is located in the N-terminal region of SaERF2 (Supplemental Fig. 3). Our observation that the GCC box-associated transactivation through *SaERF1* expression was not strongly suppressed by concomitant expression of a CMII-2 repressor-mutant of SaERF2 (ERF^CM^) (Fig. 5) confirmed that the CMII-2 at the N-terminal acts as a competitive suppressor domain.

Based on the results of our experiments using JA and ethylene inhibitors (Fig. 4a), we propose that jasmonate and ethylene signaling may be required for transcriptional activation of *SaERF1* and *SaERF2*. However, MeJA administration alone was able to activate *SaERF2* in S2 leaves, while ethylene alone upregulated *SaERF1* in S1 leaves (Fig. 4b). Thus, there are differences of the sensitivity/responsiveness of S1 and S2 accessions to these hormones. This supports the possibility that there is preferential activation of *SaERF2* in S2 by JA signaling and of *SaERF1* in S1 by ethylene signaling after MD (Fig. 2). Nontheless, it should be emphasized that biosynthesis of those hormones is not directly linked to the transcriptional changes (Fig. 3). In jasmonate signaling, an array of signal components which fine-tune plant sensitivity to the hormone, e.g., JAZ (JASMONATE-ZIM DOMAIN) repressors, JAV1 (JASMONATE-ASSOCIATED VQ-MOTIF GENE 1) and JUL1 (JAV1-ASSOCIATED UBIQUITIN LIGASE 1), function downstream of jasmonate biosynthesis in the model Arabidopsis plant^30–32^. Similarly, a suite of ethylene signaling factors such as ethylene receptors (e.g., ETR1), a protein kinase (CTR1), Nramp-like protein (EIN2) and TFs (EIN3 and EIL1) are involved in ethylene signaling and responses^33^. In *S. altissima*, the effects and regulation of these factors may be involved in transcriptional machineries that work differently according to specific genotypic variations. In addition, trans-acting factors (abbreviated here as TFs), e.g., MYBs, WRKYs and/or ERFs themselves, expressed differently between wounded S1 and S2 leaves may be involved in these differences (Supplemental Tables 1 and 2). However, specific TFs involved remain to be identified.

The possible candidates of downstream genes of SaERFs are not only defensin genes (like Arabidopsis *PDF1.2*; Fig. 6a) but also genes involved in the biosynthesis of anti-herbivore metabolites such as volatile terpenoids, because (i) putative genes involved in terpenoid synthases were also differently expressed between damaged S1 and S2 leaves (Supplemental Table S3) and (ii) our preliminary data supported that volatile organic compounds, mainly consisted of volatile terpenoids, released from S1 plants in response to *S. litura* damage were higher than those from the other clones (Rim *et al*. unpublished data). In the light of this, the nature of terpenoids that make genetic variation of *S. altissima* on their anti-herbivore abilities has been proposed in the previous studies^6,8^. However, not only ERFs but also the other member of TFs (see Supplemental Table 1) ought to be concomitantly involved in the transcriptional machinery of terpenoid synthesis genes in plants^34–38^.

Finally, we must consider the fact that at least S1-3 originated from very proximate habitats in similar ecosystems and environments. Considering the neutral theory of molecular evolution^39^, it would be necessary to account for the possibility that intraspecific polymorphism has not been extensively acquired for the environmental adaption of *S. altissima* at least 100 years after importation of *S. altissima*. Rather, such polymorphism might help to increase the fitness of the species in cases when the ecosystem and environmental conditions are drastically changed, e.g., by serious pest invasion due to global environmental changes, in the future. ERFs may contribute in part to such fitness.

## Methods

### Cloning and cultivation of *S*. *altissima*

We collected *S. altissima* plants from different sites in Shiga prefecture, Japan (S1 [35.19 N, 136.08 E], S2 [35.04 N, 136.04 E], and S3 [34.87 N, 136.06 E]) in April 2008, and collected another two *S. altissima* plants from Kumamoto prefecture and Kanagawa prefecture, Japan, in August 2016 (A1 [32.57 N, 131.12 E] and C1 [35.95 N, 139.23 E], respectively). In accord with the fact that *S. altissima* plants form an underground rhizome that sprouts multiple ramets to propagate their clones^40^, each plant genotype was propagated by repeatedly dividing the rhizomes into new pots regularly at yearly intervals. Accordingly, rhizomes collected from a single plant were divided into rhizome segments to propagate genotypic replicates. The plants were grown in soil for about 1 month after rhizomes were planted in climate-controlled rooms at 24 ± 1 °C with a photoperiod of 16 h (80 µE m^−2^ s^–1^). The potted plants (15–20 cm tall aboveground) were used for assays.

### Measurement of photosynthetic electron flow

Plants were dark-adapted for 20 min before chlorophyll fluorescence measurements. Measurements were made at 24 ± 1 °C on the upper surface of fully developed leaves (7–8 cm length) using a photosynthesis yield analyzer (MINI*-*PAM, Walz, Effeltrich, Germany).

### Larval growth assays

*S. litura* (Fabricius) (Lepidoptera: Noctuidae) was transferred to our laboratory in 2014 from a culture reared at the Sumika Technoservice Co. Ltd. (Takarazuka, Japan). The insects were reared on artificial diet (Insecta LF, Nihon Nosan Kogyo Ltd., Tokyo, Japan) in the laboratory at 24 ± 1 °C with a photoperiod of 16 h.

Third-instar *S. litura* larvae were weighed, and a larva with weight ranging from 1.8 to 2.3 mg was reared on a potted plant placed in a mesh-covered plastic box (1 L) in a climate-controlled room at 24 ± 1 °C with a photoperiod of 16 h for 2 days or 4 days. We did not use the data when a larva was dead or lost during the assays.

### Foliage damage and chemical treatment

For mechanical damage [MD] treatment, four leaves of each potted plant were subjected to removal of 1/3 of their length by clipping with scissors. Afterwards, plants were incubated in climate-controlled rooms at 24 ± 1 °C under the light condition 80 µE m^−2^ s^–1^ for 30 min or 120 min.

For MeJA treatment, potted plants were evenly sprayed with 1 mL of aqueous solution (0.1% ethanol) of MeJA (0.5 mM; Wako Pure Chemical Industrials, Ltd., Osaka, Japan) and incubated for 2 h.

Ethylene treatment was performed with cut leaves inserted in 45 mL glass vials sealed with silicon plugs. Leaves were incubated for 6 h after cutting to allow the damage response to decrease from the initial strong response to a more stable level, and then the vials were opened and ventilated for 10 min to remove any retained wound-induced ethylene. After re-closure, ethylene was applied at final concentration 1 ppm by injection through silicon plugs, and leaves were incubated for an additional 2 h before harvest.

For inhibitor treatment, four leaves of *S. altissima* plants were evenly sprayed with 2 mL per leaf of an aqueous solution of salicylhydroxamic acid (SHAM; 0.5 mM, Tokyo Chemical Industry Co., Ltd., Tokyo, Japan) or silver thiosulfate (STS; 0.5 mM, sodium thiosulfate mixed with silver nitrate, Wako Pure Chemical Industrials, Ltd.) 24 h before MD treatment. Leaves sprayed with 2 mL of water served as a mock treatment control.

### Primers

Primers used for all of the polymerase chain reactions (PCRs) in this study are listed in Supplemental Table 4.

### RNA and genome DNA isolation

Total RNA was isolated and purified from 100 mg of leaf tissues using Sepasol®-RNA I Super G (Nacalai Tesque, Kyoto, Japan) following the manufacturer’s protocol. Genomic DNA was extracted from leaves following the cetyltrimethylammonium bromide (CTAB) method^41^.

### RNA-Seq

Total RNA was purified, using a Qiagen RNeasy Mini Kit and an RNase-Free DNase Set (Qiagen, Hilden, Germany), to the following sample conditions: RNA concentration of 250 ng/µL, RIN (RNA integrity number) of > 6.5, and 28 S/18 S of > 1.0. Following purification of mRNA from total RNA (about 40 µg) using poly-T oligo-attached magnetic beads, the mRNA was fragmented into small pieces using divalent cations at elevated temperature. Illumina libraries from the above-described fragmented RNA (∼200 bp) were prepared at the core sequencing facilities at the Beijing Genomics Institute (BGI)-Shenzhen, Shenzhen, China (http://www.genomics.cn). Sequence analysis was performed using the HiSeq. 2000 system, with pair-end (2 × 90-bp) reads.

Data analysis was performed according to the method described by Ozawa *et al*. (2017)^42^. The sequences from the Illumina sequencing were deposited in DDBJ (accession number: DRA004434).

### Cloning of full-length cDNA of *SaERFs*

Because no full-length clone of the *SaERF1* cDNA sequence was available, we obtained the 3′-end sequence of *SaERF1* by RACE-PCR from S1 leaf total RNA using a first-choice RLM RACE Kit (Ambion, Foster City, CA, USA) according to the manufacturer’s protocol.

To determine the sequence of the 5′-end of the *SaERF1* cDNA, genomic DNA from S1 leaves was digested with *Xba* I and *Xho* I, and then circularized with T4 DNA ligase (Takara Bio, Kusatsu, Japan). Nested inverse PCR was performed with circularized genomic DNA as the template, using KOD -Plus- Ver.2 DNA polymerase (Toyobo, Osaka, Japan) with a pair of primers that introduced restriction enzyme cleavage sites. PCR protocol: 35 cycles of 5 s at 94 °C, 1 s at 55 °C, and 60 s at 68 °C. The resultant PCR products were subcloned and sequenced.

Finally, the ORFs of *SaERF1* and *SaERF2* cDNAs were amplified using total RNA from S1 and S2 leaves as cDNA template, respectively, using ReverTra-Plus-TM and KOD -Plus- Ver.2. Sequence data can be found in the GenBank/EMBL data libraries under accession numbers LC424188 and LC424189 for *SaERF1* and *SaERF2*, respectively.

### Mutagenesis

Mutagenesis deletion of SaERF2 from Asp^15^ to Pro^20^ (ERF2^CM^) was made by inverse PCR using PrimeSTAR® Max DNA Polymerase (Takara Bio), primers and *SaERF2* cDNA as template.

### AFLPs

After 250 ng of genomic DNA from *S. altissima* leaves was double digested with *Eco*R I and *Mse* I, the product was ligated to adapters (5′-CTCGTAGACTGCGTACCAATT-3′ and 5′-GACGATGAGTCCTGAGTA-3′). Preselected polymerase chain reaction (PCR) products were amplified using the adaptor-ligated DNA and a pair of *Eco*R I-A and *Mse* I-C primers (Supplemental Table 4). The reaction profile was 20 cycles of 20 s at 94 °C, 15 s at 56 °C, and 120 sec at 72 °C; and then a final extension for 30 min at 60 °C.

AFLPs were generated by selective PCR. For selective PCR, the *Eco*R I primer was labeled with FAM attached at the 5′ terminal. Selective primers were complementary to *Eco*R I and *Mse* I adapters, except for the addition of three selective bases at the 3′ end of both adapters to define the specificity of the selective amplification. The reaction profiles consisted of 10 cycles of 15 s at 94 °C, 15 s at the annealing temperature as it was lowered from 66 °C to 56 °C by 1 °C, and 120 s at 72 °C; 20 cycles of 10 cycles of 15 s at 94 °C, 15 s at 56 °C, and 120 s at 72 °C; and then a final extension for 30 min at 60 °C. AFLP analysis was carried out with a combination of two *Eco*R I (*Eco*R I-ACA and E-AGT) primers and two *Mse* I (*Mse* I-CTC and *Mse* I-CTA) primers (Supplemental Table 4).

PCR product size was determined using the Applied Biosystems 3730xl DNA Analyzer (Foster City, CA, USA). GeneSaAn^TM^ was used to visualize AFLP bands, which were sized by comparison to a 500-LIZ ladder added to each lane: bands <50 bp in length and bands with peak heights <500 relative fluorescent units were not scored. The data matrix was obtained for the presence (1) or absence (0) of the polymorphic fragments identified using GeneMapper^®^ ver. 4.0. Genetic distance (Nei’s original measures of genetic identity and genetic distance^43^) was measured from genome samples isolated from five independent individuals and a diagram was made using PopGene ver. 1.32.

### cDNA synthesis and quantitative PCR (qPCR)

First-strand cDNA was prepared and qPCR was performed according to the method described previously^44^. Relative transcript abundances were determined after normalization of raw signals with the abundance of the housekeeping transcript of the *S. altissima* histone gene (CL2599.Contig7; DDBJ accession number: DRA004434) or the Arabidopsis *ACT8* gene (At1g49240). We did not use samples or data when sufficient amounts of RNA were not isolated from leaves or when abnormal quantification cycle (Cq) values for the actin gene were obtained.

### Protoplast preparation and transfection, and luciferase assay

The ORF of *SaERF1* or *SaERF2* was cloned into the p35SΩ-GW-NOST vector (35SP::Ω sequence [translation enhancer]::the Gateway cassette [GW] region:: *nopaline synthase* terminator [NOST]^45^) using the Gateway cloning system (Thermo Fisher Scientific, Waltham, MA, USA). A four repeat sequence of a GCC box (AGCCGCC) fragment or a DRE element (ACCGAC) fragment was fused to a minimal TATA box::a Fluc reporter gene::NOST in the pMA cloning vector (Thermo Fisher Scientific). The map of all the vectors used is shown in Fig. 5a.

Protoplast isolation from Arabidopsis leaves and polyethylene glycol-mediated DNA transfection were performed as previously described^46,47^. The protoplast suspension (100 µL) was supplemented with a mixture of vectors carrying GCC box or DRE element::TATA::Fluc::NOST, 35SP::SaERF (SaERF1 or SaERF2)::NOST, (35SP::SaERF2 or SaERF^CM^::NOST), and reference (35SP::Renilla luciferase [Rluc]::NOST) vector at a ratio of 5:5:(5):1 to protoplast suspension with 110 µL PEG solution [40% (w/v) polyethylene glycerol, 0.4 M mannitol, and 0.1 M Ca(NO_3_)_2_4H_2_O]. The transfection was carried out at room temperature for 5 min and stopped by adding 400 µL of W5 solution. The protoplasts were collected by centrifugation at 100 *g* for 2 min and resuspended with 500 µL of WI solution (5 mM MES [pH 5.7], 0.4 M mannitol, and 20 mM KCl) and incubated in a 12-well tissue culture plate at room temperature overnight. The luciferase assay was performed as previously described^12^.

### JA, JA-Ile, ABA and SA measurements

*S. altissima* leaves (60–100 mg fresh weight) were harvested and immediately frozen in liquid nitrogen. Using 2 mL screw cap microtubes (Sarstedt, Tokyo, Japan), the samples were homogenized in FastPrep®−24 (MP Biochemicals, Santa Ana, CA, USA) using five 2.3 mm zirconia beads and 1 mL of ethyl acetate solvent spiked with deuterated internal standards (10 ng d3-JA, 5 ng d3-JA-Ile, 10 ng d6-ABA, and 20 ng d4-SA). The hormone analysis was performed according to the method described previously^48^, with slight modifications.

### Ethylene measurements

Ethylene was measured in headspace (45 mL) of 3–5 cut leaflets treated with additional MD (5 mm side incisions by scissors, 5 on each lamina part), or left without MD. Ethylene was allowed to accumulate for 6 h at normal photoperiod, after which 1 mL of headspace air was removed by syringe with needle inserted through a silicon plug. Air samples were introduced to gas chromatograph GC-2014 (Shimadzu, Kyoto, Japan) equipped with Shincarbon ST stainless steel column (length 2 m; ID 3.0 mm; SHINWA Chemical Industries, Ltd., Kyoto, Japan) via manual injection port kept at 200°C. Detector was flame ionization (FID) held at 210°C. Column was kept at constant temperature 200°C and helium flow 50 mL min^−1^. Peak area was compared to ethylene concentration obtained from external 0–1 ppm calibration curve of ethylene standard (GL Sciences Inc., Tokyo, Japan).

### Generation and cultivation of transgenic Arabidopsis plants

The ORFs of *SaERF1* and *SaERF2* cDNAs were inserted into binary vector pMDC32 (2×35SP::GW region:: NOST) using the Gateway cloning system (Thermo Fisher Scientific). The resulting vectors, pMDC32-SaERF1, pMDC32-SaERF2 or pMDC32 [vector control], were transformed into *Agrobacterium tumefaciens* strain EHA105 by electroporation. Col-0 WT Arabidopsis plants that had been grown for 6–7 weeks were transformed via the floral-dip transformation method^49^. Transgenic T_1_ seeds from each transformant were tested for germination on 1/2 Murashige and Skoog (MS) medium supplemented with 30 mg L^–1^ hygromycin. T_2_ seeds were harvested from each individual. T_2_ plants that showed a segregation ratio of about 3:1 were tested for hygromycin-resistance again. WT and T_3_ homozygous plant lines were grown in soil in climate-controlled rooms at 22 ± 1 °C with a photoperiod of 12 h (80 µE m^–2^ s^–1^) for 4 weeks and used for analyses.

### Statistical analysis

We performed one-way ANOVA with Holm’s sequential Bonferroni post-hoc test or Tukey’s HSD test using the program (http://astatsa.com/OneWay_Anova_with_TukeyHSD/) for comparing multiple samples.

## Supplementary information

Supplemental Figures.

Supplemental Table 1.

Supplemental Table 2.

Supplemental Table 3.

Supplemental Table 4.
