# Supplementary material for: Evidence that ERF transcriptional regulators serve as possible key molecules for natural variation in defense against herbivores in tall goldenrod
Source: Sci Rep. 2020 Mar 24;10:5352. doi: 10.1038/s41598-020-62142-4 (PMC7093551; doi:10.1038/s41598-020-62142-4)
Supplement: Supplementary file 1 — Supplemental Figures. [file 41598_2020_62142_MOESM1_ESM.pdf]

Evidence that ERF transcriptional regulators serve as possible key molecules for natural variation in defense against herbivores in tall goldenrod

Kento Takafuji[1], Hojun Rim[1], Kentaro Kawauchi[1], Kadis Mujiono[2,3], Saki Shimokawa[1], Yoshino Ando[4], Kaori Shiojiri[5], Ivan Galis[2], and Gen-ichiro Arimura[1]

[1] Department of Biological Science and Technology, Faculty of Industrial Science and Technology, Tokyo University of Science, Tokyo 125-8585, Japan

[2] Institute of Plant Science and Resources (IPSR), Okayama University, Kurashiki 710-0046, Japan

[3] Faculty of Agriculture, Mulawarman University, Samarinda 75119, Indonesia

[4] Field Science Center for Northern Biosphere, Hokkaido University, Sapporo 060-0809, Japan

[5] Faculty of Agriculture, Ryukoku University, Otsu 520-2194, Japan

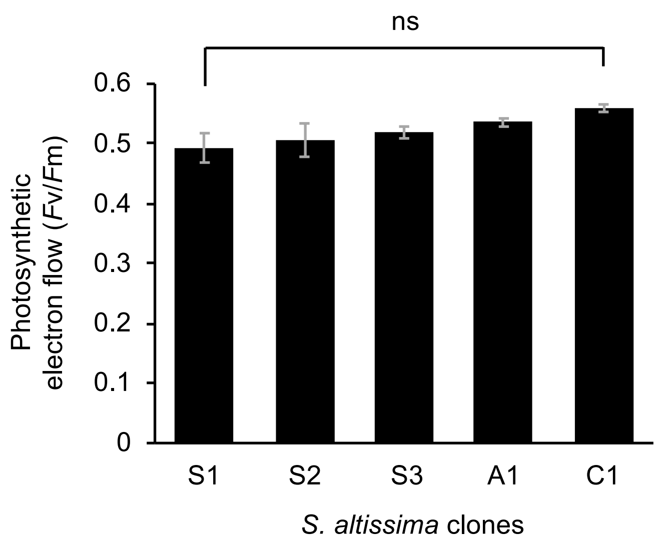

Supplemental Figure 1

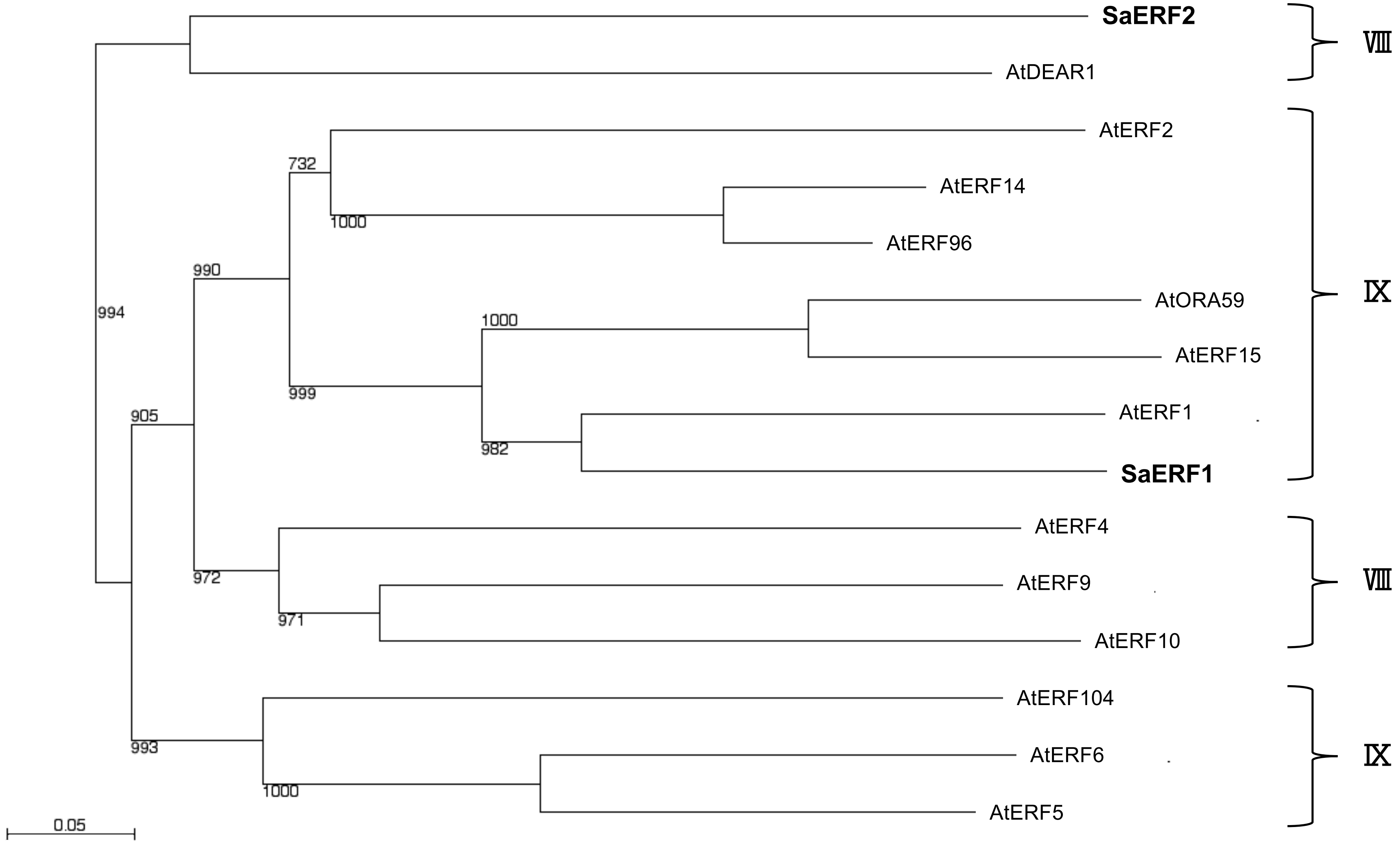

Supplemental
Figure 2

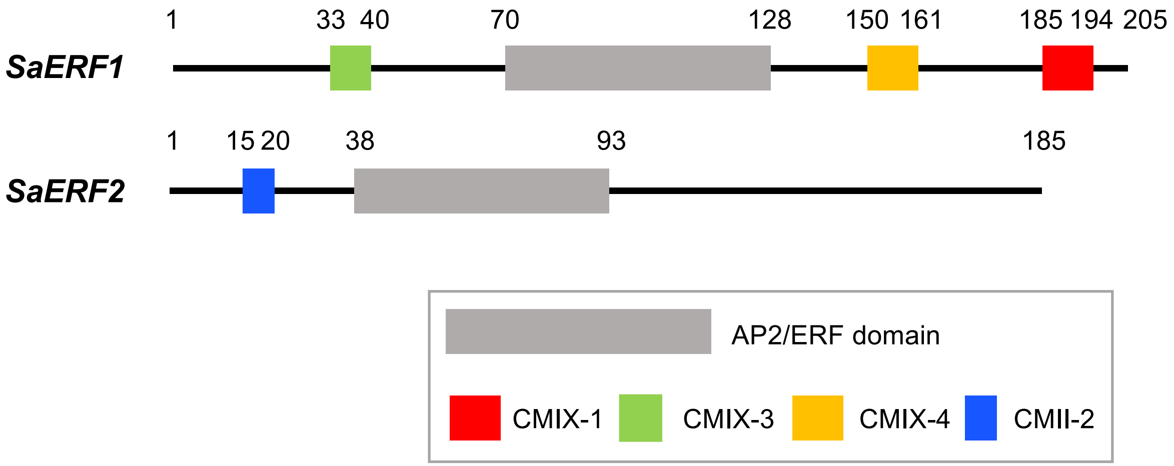

Supplemental Figure 3

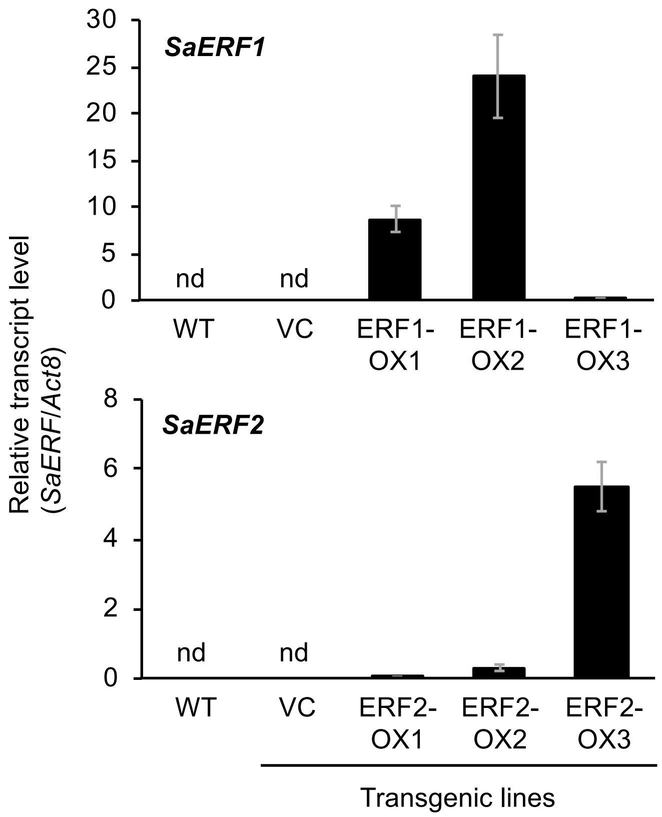

Supplemental Figure 4

Supplemental figure legends

Supplemental Figure 1. Photosynthesis activity in leaves of *Solidago altissima* clones. Photosynthesis activity on the upper surface of fully developed leaves was determined by measurement of photosynthetic electron flow.

Supplemental Figure 2. Phylogenetic trees of deduced amino acids of SaERFs and ERF group VIII and IX genes from Arabidopsis. The ERF family is grouped based on Nakano et al. (2006)[1].

Supplemental Figure 3. Schematic representation of SaERF proteins with their conserved domains.

Supplemental Figure 4. *SaERF1* and *SaERF2* expression levels in the leaves of wild-type (WT) Arabidopsis plants or their vector control (VC), *SaERF1*-expressing lines (ERF1-OX1-3), and *SaERF2*-expressing lines (ERF2-OX1-3). Transcript levels of genes were measured by RT-qPCR, and relative transcript abundances of *SaERF* were determined after normalization of raw signals with the abundance of the housekeeping transcript of the Arabidopsis *ACT8* gene (At1g49240). Data represent the mean ± standard error (n = 4-5). nd, not detected.

Reference

1 Nakano, T., Suzuki, K., Fujimura, T. & Shinshi, H. Genome-wide analysis of the ERF gene family in Arabidopsis and rice. *Plant Physiol.* **140**, 411-432, doi:10.1104/pp.105.073783 (2006).
